# Dynamic Plasma KRAS ctDNA Profiling Identifies Treatment Response and Nascent Resistance in Metastatic Non-Small-Cell Lung Cancer: A Pilot Study

**DOI:** 10.3390/cancers18183009

**Published:** 2026-09-16

**Authors:** Valentina Buzzi, Greta Berti, Michele Santillo, Arianna Belli, Elisabetta Rosi, Giulia Favarato, Federico Lavorini, Sara Tomassetti, Annarosa Arcangeli, Mauro Iannopollo, Elena Lastraioli

**Affiliations:** 1 Department of Experimental and Clinical Medicine, University of Florence, 50121 Florence, Italy; v.buzzi@student.unisi.it (V.B.); michele.santillo@unifi.it (M.S.); federico.lavorini@unifi.it (F.L.); sara.tomassetti@auslromagna.it (S.T.); annarosa.arcangeli@unifi.it (A.A.); 2 Department of Medical Biotechnologies, University of Siena, 53100 Siena, Italy; 3 Medical Oncology, S. Jacopo Hospital, 51100 Pistoia, Italy; greta.berti@uslcentro.toscana.it (G.B.);; 4 Medical Oncology, S.S. Cosma e Damiano Hospital, 51017 Pescia, Italy; 5 Pulmonology and Intensive Care Unit, Careggi University Hospital, 50134 Florence, Italy; arianna.belli@unifi.it (A.B.); rosiel@aou-careggi.toscana.it (E.R.); 6 Interventional Pulmonology Unit, Careggi University Hospital, 50134 Florence, Italy; giulia.favarato@unifi.it

**Keywords:** liquid biopsy, lung cancer, *KRAS*, therapy monitoring

## Abstract

**Background:** *KRAS* mutations are common oncogenic drivers in metastatic lung cancer (mLC) and may have prognostic and therapeutic implications. Circulating tumor DNA (ctDNA) analysis offers a minimally invasive approach for detecting tumor-specific alterations and monitoring tumor dynamics. This pilot study investigated the concordance between tissue and plasma *KRAS* mutational status and evaluated its clinical significance. **Methods:** Forty patients with mLC (25 males, 15 females; mean age 70.8 years) were included. *KRAS* mutations were analyzed in formalin-fixed paraffin-embedded tumor tissue using next-generation sequencing and in baseline plasma ctDNA using the Biocartis Idylla™ system. Concordance was assessed using Cohen’s kappa statistics. Associations with clinicopathological characteristics and survival outcomes (overall survival (OS), progression-free survival (PFS), and time to progression (TTP)) were examined. Longitudinal ctDNA monitoring was performed in a subset of patients. **Results:** *KRAS* mutations were detected in 14/36 (38.89%) of tumor tissues and 12/40 (30.00%) of plasma samples, predominantly involving codon 12, with G12C as the most frequent variant. Tissue–plasma concordance was assessed in the 36 patients with matched samples and was substantial (*κ* = 0.64), while mutation subtype showed almost perfect agreement among concordant cases (percentage of agreement: 88.9%, *κ* = 0.80, *p* = 0.002, 95% CI: 0.4400–1.0000). No significant associations were observed with clinicopathological variables. Longitudinal analysis revealed dynamic ctDNA changes, with molecular progression occasionally preceding clinical progression. Patients with *KRAS*-mutated ctDNA had shorter PFS and OS compared to those with wild-type ctDNA (median PFS: 134 vs. 146 days; median OS: 163 vs. 193 days). **Conclusions:** Plasma ctDNA detection of *KRAS* mutations identifies a poor-prognosis subgroup of mLC patients and provides dynamic insights into tumor evolution. Serial ctDNA monitoring may anticipate disease progression and support real-time assessment of treatment response, highlighting its potential role in translational research and precision oncology.

## Simple Summary

Mutations in the *KRAS* gene are common in metastatic lung cancer and may influence how the disease behaves and responds to treatment. In this study, we compared *KRAS* mutations detected in tumor tissue with those found in blood samples using circulating tumor DNA (ctDNA), a minimally invasive “liquid biopsy” method. We found moderate agreement between tissue and blood tests, but when the same mutation was detected, the results were fully consistent. Patients with *KRAS* mutations detected in blood had shorter survival and faster disease progression. In some cases, changes in ctDNA appeared before clinical signs of progression, suggesting that blood-based monitoring could help track disease in real time and improve patient management.

## 1. Introduction

Lung cancer (LC) is among the most frequently diagnosed and lethal tumors worldwide. According to the most recent estimates, LC caused approximately 1.8 million deaths and accounted for about 2.4 million new cases in 2022 (Source: Globocan, https://gco.iarc.fr, accessed on 21 February 2026, [1,2]). LC incidence is higher in high-income countries, largely because tobacco smoking remains the principal risk factor [3,4,5,6]. Histologically, LC is divided into two main types: small-cell lung cancer (SCLC) and non-small-cell lung cancer (NSCLC). NSCLC is further subclassified into lung adenocarcinoma (LUAD), squamous cell carcinoma (LSCC), large cell carcinoma, and bronchial carcinoid tumor; LUAD is the most common NSCLC subtype and one of the most frequent primary malignancies. LC pathogenesis involves both genetic alterations (point mutations, deletions, insertions, and duplications) and epigenetic changes (DNA methylation, histone deacetylation, and microRNA dysregulation) [7,8,9]. Recently, tissue- and blood-based biomarkers—including circulating tumor cells (CTCs), circulating tumor DNA (ctDNA), microRNAs (miRNAs), and exosomes—have improved diagnosis and treatment selection for patients with advanced NSCLC [10,11,12,13,14] as well as preneoplastic conditions [15] and rare diseases [16,17]. Apoptotic and necrotic cells are the main source of circulating cell-free DNA (cfDNA) and total cfDNA levels are elevated in many cancers, including LC [11]. In the oncology field, the levels of cfDNA derived from tumor cells (ctDNA) are particularly relevant, and ctDNA-based assays for clinically actionable alterations (for example, *EGFR*, *NRAS*, *KRAS*, and *BRAF* mutations) have also been approved for use in NSCLC [18]. *KRAS* mutations occur primarily in the LUAD histotype and have a higher frequency in smokers [19]. Also in LC, *KRAS* mutations are associated with worse outcomes due to the acquired resistance to epidermal growth factor receptor (*EGFR*) inhibitors [20]. Activating *KRAS* mutations occur predominantly at codon 12 (notably G12C, G12D, and G12V), resulting in a constitutively active RAS protein, thus driving proliferation independently of upstream receptor signaling; major downstream effectors include the MAPK/ERK, PI3K/AKT, and RalGDS pathways [21]. The *KRAS* G12C variant is the most prevalent, accounting for roughly 45% of *KRAS* mutations in NSCLC, and it is the most strongly associated with poor outcome [22,23]. Moreover, *KRAS* activating mutations (and in particular the G12C mutation) can represent a target for therapy with novel agents such as Adagrasib and Sotorasib [24]. *KRAS* mutations can be detected either in tissue samples or in plasma, and in different clinical settings, it has been shown that the mutations carried by ctDNA hold valuable prognostic potential in different kinds of tumors, as witnessed by several original papers as well as systematic reviews and meta-analyses [25,26,27,28,29,30,31].

The aims of this pilot study were the following: (1) to evaluate the concordance between *KRAS* mutational status in tumor tissue and plasma in a cohort of patients with metastatic lung cancer; (2) to monitor patients at different time points during treatment to establish eventual associations between the presence (and levels) of mutated clones and best response; and (3) to assess eventual associations between *KRAS* mutational status and clinical, pathological parameters or survival.

## 2. Materials and Methods

### 2.1. Study Design, Population, and Setting

The study reported herein was a biological, observational, prospective, multicenter investigation that involved the collection of blood specimens and clinical data from 40 patients with metastatic lung cancer (mLC) classified as NSCLC receiving treatment for metastatic disease, followed up at S. Jacopo Hospital (Pistoia, Italy), S.S. Cosma e Damiano Hospital (Pescia, Italy), and the Pneumology and Respiratory Intensive Care Unit of Careggi University Hospital (Florence, Italy) between December 2023 and February 2026. Eligible patients had a histologically confirmed TNM stage IV lung carcinoma (staged through the AJCC version 8 [32], were treatment-naïve, and had measurable disease according to Response Evaluation Criteria in Solid Tumors (RECIST v1.1 released in 2009 by the RECIST Working Group) [33]. The protocol (ID 25189_bio) was approved by the local ethics committee, released on 29 November 2023 for Pistoia and Pescia and on 2 February 2025 for Careggi, and all participants provided written informed consent at enrolment in accordance with the Declaration of Helsinki.

### 2.2. Patient Assessment and Follow-Up

Demographic, clinical and treatment data were extracted from clinical records at enrolment.

In clinical practice, lung cancer patients received chemotherapy and targeted or biological therapies as indicated. First-line treatment continued until radiological progression or unacceptable toxicity. Computed tomography scans were obtained at baseline (prior to initiating first-line therapy) and every three months thereafter to monitor response according to clinical standards. All-cause mortality was recorded prospectively. The median overall survival was 163 days for *KRAS*-mutated patients and 193 days for wild-type *KRAS*. The median progression-free survival PFS was 134 days in mutated patients and 146 days in wt *KRAS*.

### 2.3. Tissue Histopathology, Quality Control, and NGS Sequencing

Tumor tissue status had been previously assessed on formalin-fixed, paraffin-embedded (FFPE) biopsies from primary lesions or metastases using next-generation sequencing (NGS) performed by experienced laboratory personnel at the participating hospitals as part of routine diagnostics.

FFPE tissue blocks were sectioned and evaluated by experienced pathologists as a routine procedure. Tumor cell fraction (tumor purity) was assessed on hematoxylin and eosin-stained slides, requiring a minimum threshold of 50% tumor content for further molecular analyses. Once the purity of the tumor samples was proven, genomic DNA was extracted using the QIAamp DNA FFPE Tissue Kit (Qiagen, Venlo, The Netherlands) or MagCore^®^ Genomic DNA FFPE (RBC Bioscience, New Taipei City, Taiwan) depending on the procedures followed at the institution at which patients were enrolled, according to the manufacturer’s protocol.

NGS profiling was carried out using a targeted panel (CE-IVD Myriapod^®^ NGS Cancer panel DNA or Myriapod^®^ NGS Cancer probe PLUS, Diatech Pharmacogenetics, Jesi, Italy) on the Illumina MiSeq or NextSeq platform. The mean target sequencing depth was 500×. The limit of detection (LOD) for variant allele frequency (VAF) was set at 5%. The analysis was carried out with Myriapod^®^ NGS Data Analysis Software version 5.0.10, and the ClinVar, OncoKB, My Cancer Genome, and Franklin databases were interrogated.

Tissue NGS *KRAS* status was available for 36/40 patients and baseline plasma ctDNA for 40/40; concordance analyses were thus restricted to paired cases (*n* = 36).

### 2.4. Sample Collection and Processing

Blood samples were collected at baseline (T0), prior to initiation of first-line treatment, and a subgroup of patients was subsequently monitored at 4, 8, 12, and 24 weeks until disease progression or death. Approximately 10 mL of peripheral blood was drawn from each patient into K2-EDTA BD Vacutainer^®^ (BD, Franklin Lakes, NJ, USA) or Cell-Free DNA BCT (Streck, Omaha, NE, USA) tubes by nursing staff at the participating hospitals, immediately before starting therapy. Samples were delivered to the central laboratory of the Department of Experimental and Clinical Medicine and processed within 4 h for K2-EDTA tubes or within 72 h for Cell-Free DNA BCT tubes. Plasma was prepared using a standard protocol consisting of two consecutive centrifugation steps: first at 1600× *g* for 10 min at room temperature to separate plasma from cellular components, followed by 6000× *g* for 10 min at room temperature to remove residual platelets and cellular debris. Purified plasma was aliquoted into cryogenic tubes (1–2 mL per aliquot) and stored at −80 °C for long-term preservation.

### 2.5. ctDNA Analysis in Plasma Samples

The analysis was carried out through the Biocartis Idylla™ system to detect 21 mutations across codons 12, 13, 59, 61, 117, and 146 of the *KRAS* gene (IDYLLA™ ctKRAS MUTATION TEST) (Biocartis, Mechelen, Belgium) on the Idylla™ platform. This cartridge-based assay utilizes real-time PCR/digital-like detection to identify 21 *KRAS* mutations in codons 12, 13, 59, 61, 117, and 146 directly from 1 mL of plasma without requiring previous DNA extraction.

The assay was performed according to the manufacturer’s instructions. Independent analytical validation studies for the Idylla™ ctKRAS assay have established an analytical specificity > 99% and an LOD < 1% VAF (reaching 0.1% for prevalent codon 12/13 substitutions). No in-house re-validation of the platform was conducted for the present study, relying on the manufacturer’s standardized CE-IVD/RUO validation metrics.

#### Idylla™ Platform Methodology

The Idylla™ ctKRAS Mutation Assay executed on the fully automated Idylla™ system utilizes single-use, microfluidic cartridges pre-loaded with all reagents required for nucleic acid extraction, amplification, and detection. The procedure takes place as follows:

1. Sample Loading: A fixed input volume of 1 mL of plasma was pipetted directly into the dedicated sample insertion port of the cartridge without prior DNA extraction and purification.
2. Automated On-Cartridge Processing: Upon insertion into the instrument, high-intensity focused ultrasound (HIFU) and chemical lysis within the cartridge release circulating cell-free DNA (cfDNA). The extracted nucleic acids are subsequently purified via magnetic bead-based binding and eluted into internal microfluidic channels.
3. Amplification and Detection: The eluted cfDNA is distributed into multiplex real-time PCR reaction chambers bearing allele-specific primers and fluorescently labeled TaqMan™ probes. The assay covers 21 *KRAS* mutations in codons 12, 13, 59, 61, 117, and 146 (including G12D, G12V, G12C, G12A, G12S, G12R, and G13D, among others).
4. Signal Interpretation: Fluorescence signals generated during PCR cycling are automatically processed by onboard software (version 11.24.0.8) using proprietary delta cycle threshold (ΔCq) algorithms. Results are reported as qualitative mutation status (Detected/Not Detected) along with quantitative ΔCq values reflecting mutational burden relative to total circulating DNA.

Quantitative evaluation in this study was derived using the cycle threshold difference (ΔCq = Cq_control_ − Cq_mutant_). In accordance with prior analytical validation studies of the Idylla™ ctDNA platform [34,35], the assay achieves a robust LOD down to 0.1–1% VAF depending on the specific codon 12/13 mutation. Samples were categorized as ctDNA-positive based on the manufacturer’s validated algorithmic cutoff ΔCq > threshold, ensuring high analytical specificity and avoiding false-positive background noise.

### 2.6. Statistical Analysis

Diagnostic performance parameters and agreement between the detection of mutations in tissue and plasma were evaluated using a 2 × 2 contingency table in which Positive Percent Agreement (PPA/Sensitivity), Negative Percent Agreement (NPA/Specificity), Positive Predictive Value (PPV), Negative Predictive Value (NPV), and Overall Percent Agreement (OPA/Accuracy) were calculated. Two-sided 95% confidence intervals (95% CI) were estimated using the Wilson score method. Cohen’s kappa coefficient (*κ*) was determined to assess inter-assay agreement and interpreted according to Landis and Koch criteria.

Differences in marginal proportions of mutation positivity between matched tissue and plasma samples were assessed using McNemar’s exact test for paired nominal data.

Associations between clinical variables and gene expression (measured as ΔCq) were evaluated with nonparametric tests. For comparisons between two independent groups with non-normally distributed data, the Mann–Whitney U test was applied. To analyze the correlation between continuous variables, the Spearman correlation coefficient was applied. A two-sided significance level of 5% was used for all tests.

Survival analyses were performed using Kaplan–Meier estimates and the log-rank test. Overall survival (OS) was defined as the interval from diagnosis of metastatic disease to death or last follow-up. Progression-free survival (PFS) was defined as the time from baseline to the first documented radiological/clinical disease progression (according to RECIST v1.1) or death from any cause. Patients without disease progression or death at the time of analysis were censored at the date of their last tumor assessment demonstrating disease control. Time to progression (TTP) was defined as the time from treatment initiation to radiologically documented disease progression according to RECIST v1.1 criteria. Patients without progression were censored at the date of last follow-up.

Univariate and multivariate survival analyses were performed using Cox proportional hazards regression models to evaluate the independent prognostic impact of plasma *KRAS* status on OS and PFS. Hazard ratios (HRs) and 95% CIs were calculated. Variables with a *p* value < 0.10 in univariate analysis or recognized clinical prognostic relevance (age and sex) were included in the multivariate Cox model. Given the sample size (*n* = 40), parsimonious modeling was applied to prevent overfitting. Statistical significance was set at two-tailed *p* < 0.05.

Statistical analyses were conducted with GraphPad Prism version 10 (GraphPad Software, RRID: SCR_002798, San Diego, CA, USA).

## 3. Results

The cohort comprised 25 males and 15 females with a mean age of 70.8 years (range 41–84). The main demographic and clinicopathological characteristics are listed in Table 1, while the therapy schedule is reported in Table S1.

### 3.1. KRAS Mutational Status in Tissue and Plasma Samples

**Figure 1:**
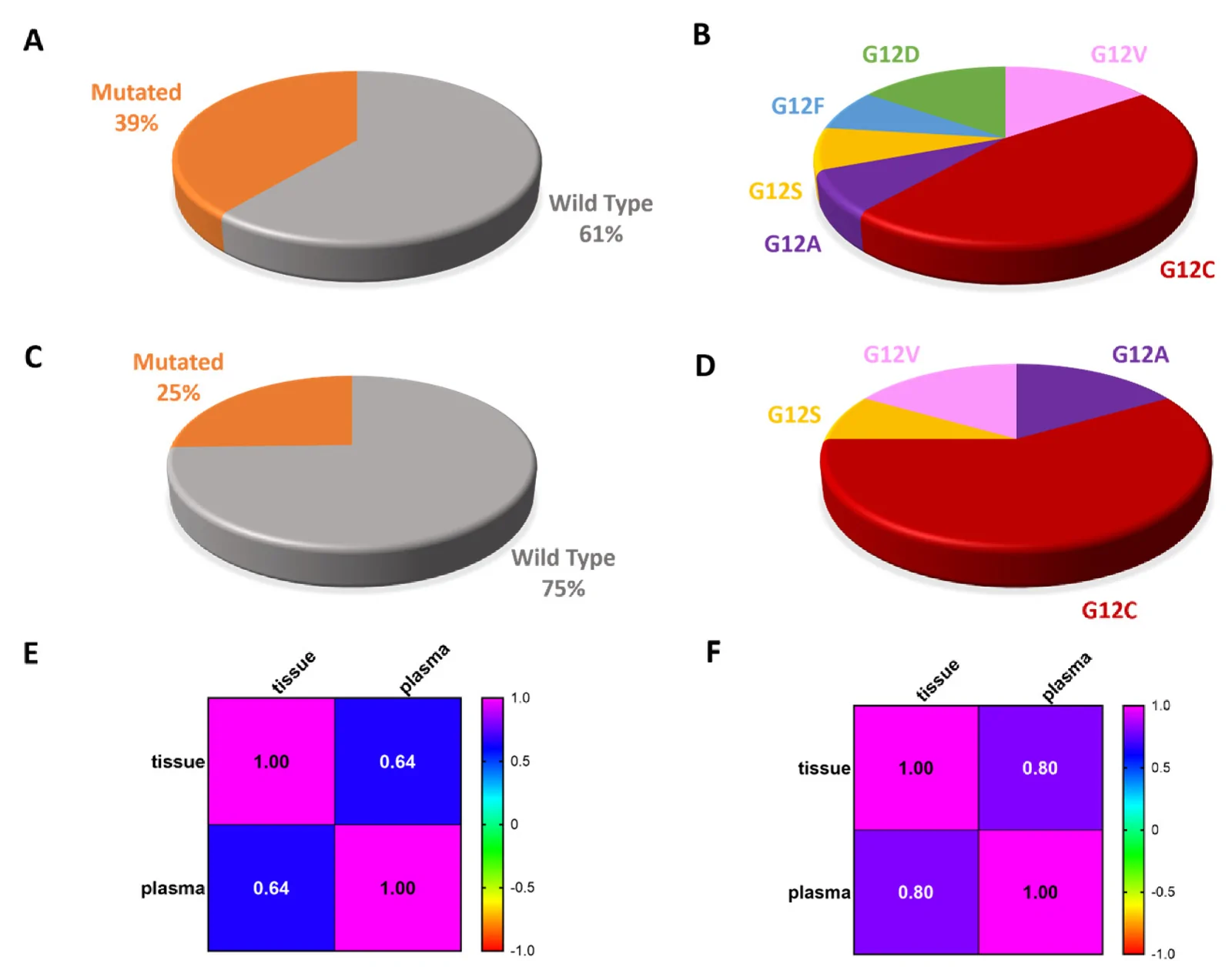
(**A**–**D**) Pie charts showing the frequency of *KRAS* status and type of mutations in tissue (**A**,**B**) and plasma (**C**,**D**). (**E**,**F**) Correlation matrices between *KRAS* status (**E**) and type of mutation (**F**) in tissue and plasma. Spearman correlation test.

Molecular analysis for *KRAS* was performed on 36 FFPE tumor tissue biopsies (by means of NGS technology), and from this evaluation, it emerged that 14 out of 36 (38.89%) patients harbored *KRAS* mutations, while 22 (61.11%) were classified as wild type (Figure 1A). Overall, the most common mutation was represented by G12C, but all the mutations detected affected codon 12 (Figure 1B).

**Figure 2:**
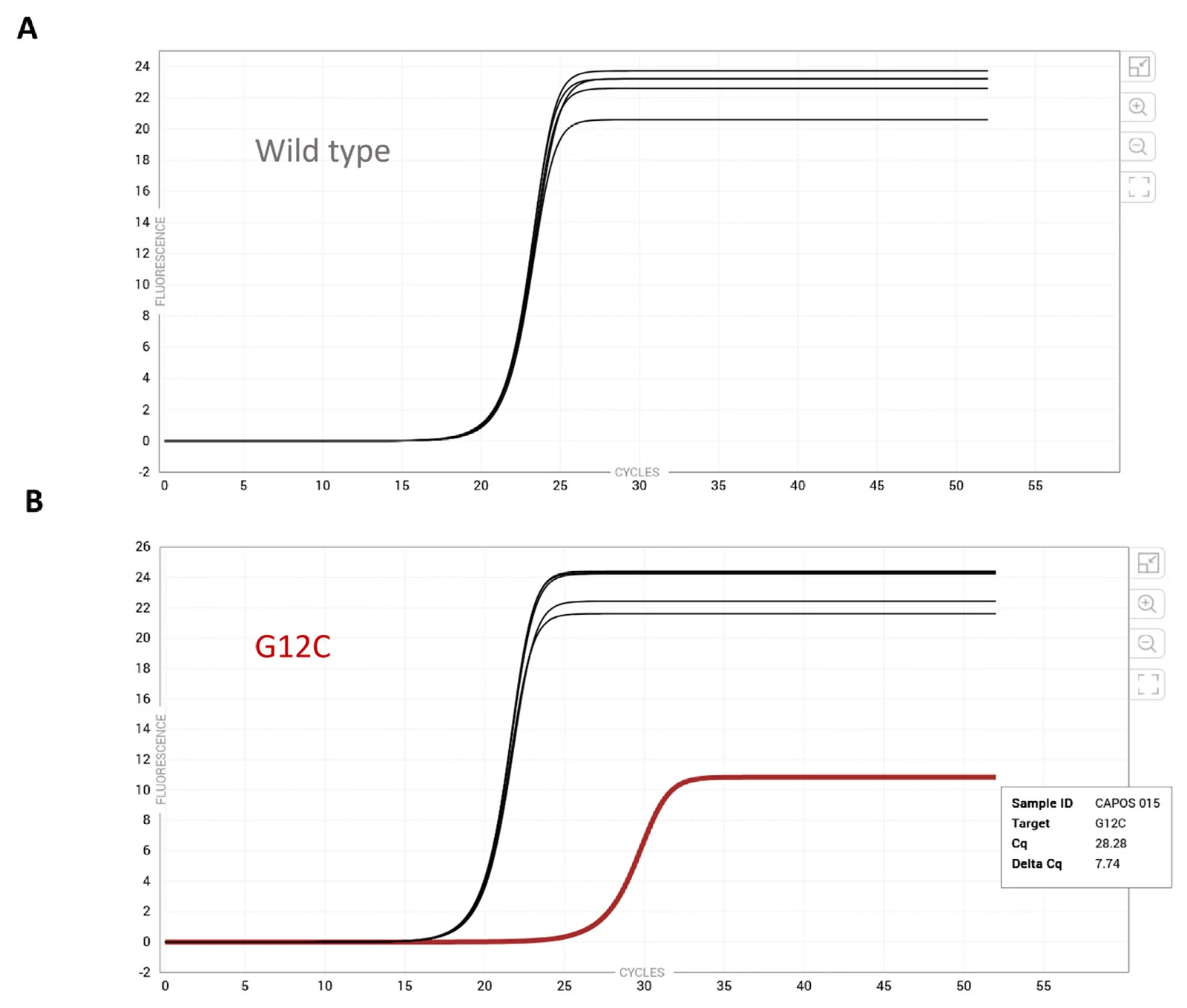
Amplification curves of *KRAS* in representative samples obtained through the Biocartis Idylla^TM^ platform. (**A**) Wild-type sample. (**B**) G12C-mutated sample.

As described in the Materials and Methods, the mutational status of plasma samples at baseline was evaluated through the Biocartis Idylla^TM^ platform. Figure 2 shows representative amplification curves of wild-type samples (Figure 2A) or samples bearing the most frequent mutation (G12C, Figure 2B). Both the wild-type and mutated samples showed exponential amplification curves, indicating successful target amplification. The wild-type sample showed earlier amplification with a Ct value of 22.63 (black curves in panel A and B), whereas the mutated sample amplified at cycle 28.28 (red curve in panel B). In addition, the mutant curve exhibited a less steep exponential phase and a lower fluorescence plateau. These differences suggest reduced amplification efficiency in the mutated template, potentially due to altered primer/probe binding caused by the mutation. Representative curves of other types of mutations are reported in Figure S1.

Overall, as shown in Figure 1C, 28 out of 40 samples (70.00%) turned out to be wild type, while 12 out of 40 (30.00%) had a mutation in the *KRAS* gene. As observed in tissue samples, the most frequent mutation was G12C, also in plasma samples (Figure 1D). Plasma ctDNA testing demonstrated an Overall Percent Agreement (OPA) of 83.3% (95% CI: 68.1–92.1%). Inter-method concordance yielded a Cohen’s kappa coefficient of 0.640 (95% CI: 0.377–0.903), indicating moderate agreement between tissue biopsy and liquid biopsy (Figure 1E and Table 2). Mutations were detected in both tissue and plasma in 10 patients, while 20 patients were negative in both assays. Four patients harbored a mutation identified in tissue but not detected in plasma, and two patients showed a plasma-positive result without a corresponding mutation in the matched tissue sample. The Positive Percent Agreement (PPA) was 71.4% (95% CI: 45.4–88.3%), while the Negative Percent Agreement (NPA) reached 90.9% (95% CI: 72.2–97.5%). The Positive Predictive Value (PPV) and Negative Predictive Value (NPV) were both 83.3% (95% CI: 55.2–95.3% and 64.1–93.3%, respectively).

Given the potential influence of the marginal distribution of positive and negative results on Cohen’s kappa, the prevalence- and bias-adjusted kappa (PABAK) was additionally calculated and was 0.667, with an overall observed agreement of 83.3% (Table 2). Assessment of discordant paired results using McNemar’s exact test revealed no statistically significant marginal asymmetry between plasma ctDNA and tissue genotyping (*p* = 0.6875). This result indicates that a statistically significant difference in the marginal proportions of positive results was not detected; however, it should not be interpreted as evidence of the absence of systematic detection bias, particularly given the limited number of discordant pairs.

Interestingly, among paired cases with tissue and plasma positivity (*n* = 10), mutation type matched in almost all cases (percentage of agreement: 88.9%, *κ* = 0.80, *p* = 0.002, 95% CI: 0.440–1.000; Figure 1F).

Quantitative correlation between tissue VAF and plasma molecular levels was evaluated in 29 patients with available matched quantitative data. A strong positive correlation was observed (Spearman’s ρ = 0.7757, 95% CI: 0.5335–0.8824, *p* < 0.0001), indicating that higher tissue mutational burden was associated with higher ctDNA levels in plasma.

### 3.2. Association of KRAS Status with Clinical Parameters

**Figure 3:**
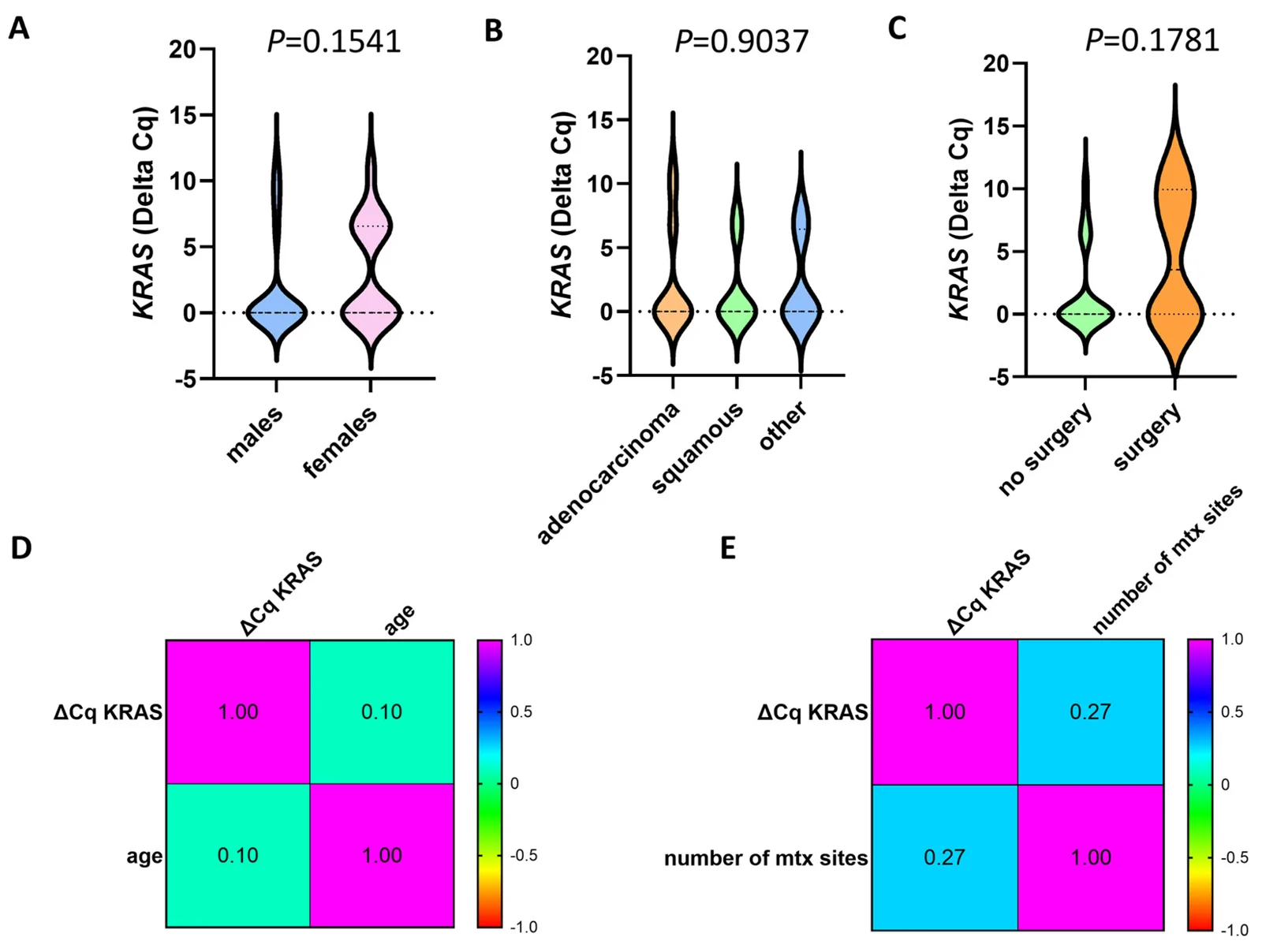
(**A**–**C**) Violin plots showing the associations between mutated *KRAS* expression levels and gender (**A**), histotype (**B**), and surgery (**C**). Mann-Whitney test. (**D**,**E**) Correlation matrices between mutated *KRAS* gene expression and patient age (**D**) and number of metastatic sites (**E**) at enrolment. Spearman correlation test.

Once the mutational profile of the patients enrolled in the study was assessed, we moved forward to evaluating the presence of an association with clinical features. As shown in the violin plots and correlation matrices in Figure 3, no statistically significant association emerged when evaluating mutated *KRAS* expression levels. However, some differences emerged between groups: the violin plots of the ΔCq values, indicating the expression levels, showed that a broader distribution was observed in females (*p* = 0.1541, Figure 3A), patients with adenocarcinomas with respect to squamous carcinoma (*p* = 0.9037, Figure 3B), and patients who underwent surgery for the primary tumor (*p* = 0.1781, Figure 3C). No statistically significant correlations were highlighted when evaluating age at enrolment (Figure 3D, *p* = 0.532) and the number of metastatic sites at enrolment (Figure 3E, *p* = 0.104), indicating the presence of a very weak (ρ = 0.10) and weak correlation (ρ = 0.27), respectively.

### 3.3. Exploratory Longitudinal Monitoring of KRAS Mutational Status During Treatment

For a subset of patients (*n* = 9), plasma *KRAS* mutational status and gene expression levels were re-evaluated approximately every four weeks from the start of therapy (at 4, 8, 12, and 24 weeks, when available, or until documented disease progression). In most patients, *KRAS* mutational status remained stable throughout treatment, whereas temporal variations were observed in some cases. ΔCq values, mutation type, treatment, and best clinical response for all longitudinally monitored patients are reported in Table 3.

Given the small number of patients undergoing serial monitoring and the observational nature of the analysis, these longitudinal data were considered exploratory and hypothesis-generating. No criteria for molecular progression or molecular response were prospectively predefined. Therefore, changes in plasma *KRAS* status and ΔCq values are described as temporal molecular changes and are not considered validated measures of treatment response or disease progression.

**Figure 4:**
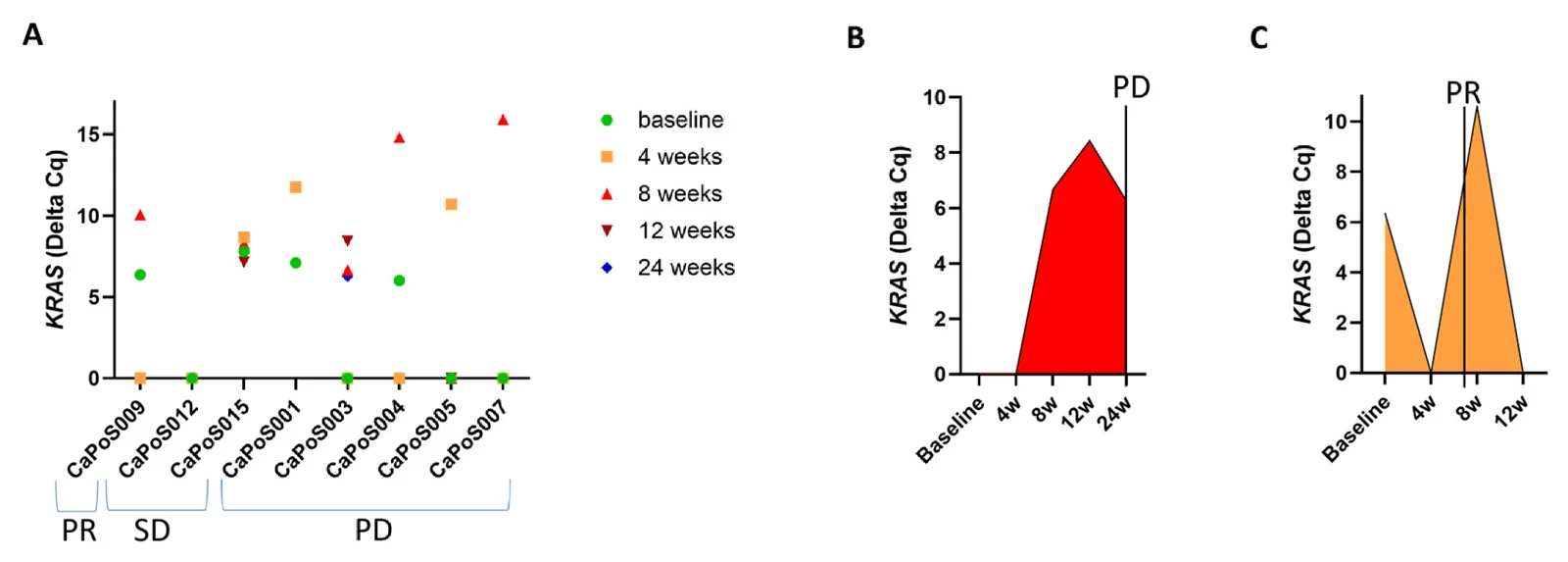
Longitudinal dynamics of plasma *KRAS* status. (**A**) Categorical scatter plot illustrating baseline and follow-up plasma *KRAS* mutation levels (ΔCq) in individual patients grouped according to best clinical response: partial response (PR), stable disease (SD), and progressive disease (PD). Time points are represented by distinct shapes and colors (baseline, green circles; 4 weeks, orange squares; 8 weeks, red triangles; 12 weeks, dark red inverted triangles; 24 weeks, blue diamonds). (**B**) Representative ctDNA kinetic profile of a patient experiencing progressive disease, illustrating a temporal increase in plasma *KRAS* signal preceding clinically documented disease progression. (**C**) Representative ctDNA kinetic profile of a patient achieving partial response, illustrating a decline in plasma *KRAS* signal preceding clinical response. These examples are descriptive and hypothesis-generating and do not establish the clinical utility of longitudinal ctDNA monitoring.

As shown in the scatter plot in Figure 4A, variations in mutated KRAS levels were observed across several patients undergoing longitudinal monitoring. In CaPoS001, the baseline G12V mutation remained detectable at 4 weeks, with an increase in ΔCq from 7.11 to 11.72, followed by progressive disease and death. In CaPoS004, the baseline G12C mutation was detected at 8 weeks together with the emergence of a second G12R mutation. The patient subsequently experienced disease progression and received second-line treatment. These observations illustrate temporal changes in plasma *KRAS* status during treatment; however, their biological and clinical significance cannot be established from this small cohort.

CaPoS003 was wild type at baseline and at the first monitoring time point (4 weeks), but a G12D mutation was detected at 8 weeks and remained detectable at 12 and 24 weeks. The ΔCq increased from 6.67 at 8 weeks to 8.44 at 12 weeks and subsequently decreased to 6.27 at 24 weeks (Figure 4B). The emergence and persistence of the *KRAS* mutation preceded clinically documented disease progression. This temporal sequence is compatible with the hypothesis that longitudinal ctDNA monitoring may capture molecular changes before clinically documented disease progression; however, the observation is descriptive and does not establish a predictive or clinically actionable role for ctDNA.

CaPoS009 displayed a fluctuating mutational pattern, with a G12S mutation detected at baseline and at 8 weeks but not at 4 or 12 weeks (Figure 4C). The transient increase in mutated *KRAS* detected at 8 weeks occurred in the context of a delay in treatment administration due to significant comorbidities. Following treatment resumption, the mutation became undetectable and the patient subsequently achieved a partial response. This temporal association is consistent with a possible relationship between ctDNA dynamics and treatment response, although causality cannot be established from a single observational case.

Overall, these longitudinal observations indicate that plasma *KRAS* status may undergo dynamic changes during treatment and, in selected patients, may change before clinically documented alterations in disease status. However, given the small number of longitudinally monitored patients, the non-standardized nature of serial sampling, and the absence of prospectively defined criteria for molecular response or progression, these findings should be regarded as hypothesis-generating. Whether longitudinal ctDNA monitoring can provide clinically actionable information or influence treatment decision-making remains to be determined in prospective studies.

### 3.4. Survival Analyses

**Figure 5:**
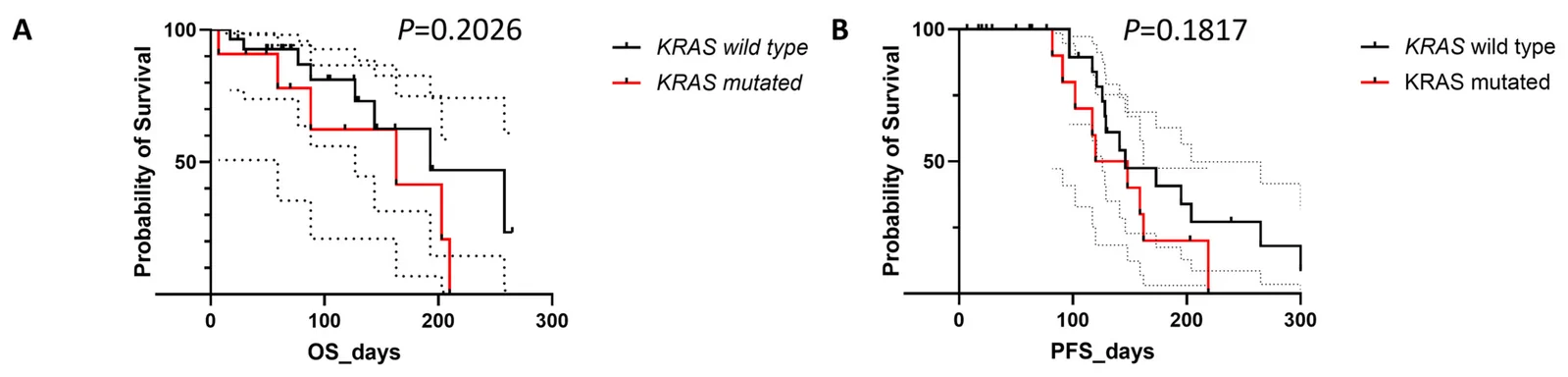
Survival analysis. (**A**) Kaplan–Meier plot of OS. (**B**) Kaplan–Meier plot of PFS. Black curves: wild-type *KRAS* patients; red curves: mutated *KRAS* patients. Dotted lines represent 95% confidence intervals.

To determine whether the presence of *KRAS* mutations in ctDNA could serve as a potential prognostic indicator, we conducted survival analyses evaluating overall survival (OS), progression-free survival (PFS), and time to progression (TTP), as defined in the Section 2 (Figure 5).

As illustrated by the red curves in Figure 5A,B, patients harboring *KRAS* mutations exhibited a poorer prognosis compared with those with wild-type *KRAS*. Patients with detectable plasma *KRAS* mutations exhibited shorter median OS compared to those with wild-type ctDNA (*p* = 0.2026; median OS: 163 vs. 193 days; 95% CI: 59–not reached for mutated *KRAS* and 127–not reached for wild-type *KRAS*) and slightly shorter PFS (*p* = 0.1817; median PFS: 134 vs. 146 days; 95% CI: 82–162 for mutated *KRAS* and 126–204 for wild-type *KRAS*).

A similar trend was observed for TTP, for which statistical significance was reached (Figure S2), although this finding should be interpreted with extreme caution due to the low number of events detected (*p* = 0.0455; median TTP: 83 vs. 147 days; 95% CI: 83 for mutated *KRAS* and 89–not reached for wild-type *KRAS*).

To confirm whether the prognostic impact of baseline plasma *KRAS* positivity was independent of potential clinical confounders, a multivariate Cox proportional hazards model was performed, incorporating age and sex as covariates (Table 4).

Baseline ctDNA *KRAS* positivity was not significantly associated with disease progression (adjusted HR = 1.902, 95% CI: 0.741–4.731, *p* = 0.176) or overall mortality (adjusted HR = 1.770, 95% CI: 0.528–5.677, *p* = 0.346). Age was not significantly associated with PFS (adjusted HR = 0.994, 95% CI: 0.941–1.051, *p* = 0.829) or OS (adjusted HR = 1.041, 95% CI: 0.961–1.147, *p* = 0.376), while sex was not significantly associated with PFS (adjusted HR = 1.233, 95% CI: 0.515–2.992, *p* = 0.636) or OS (adjusted HR = 3.892, 95% CI: 0.998–25.69, *p* = 0.101). Given the limited sample size and number of events, these multivariable models should be considered exploratory and interpreted with caution. They do not provide sufficient evidence to establish baseline ctDNA *KRAS* status as an independent prognostic factor.

## 4. Discussion

In this pilot study, we evaluated the concordance between tissue and plasma *KRAS* mutational status and explored the clinical relevance of circulating tumor DNA analysis in metastatic lung cancer. Our findings showed an overall observed agreement of 83.3% between tissue and plasma, with a Cohen’s kappa coefficient of 0.640. The PPA of 71.4% indicates that a proportion of *KRAS* alterations identified in tissue were not detected in plasma, which may reflect biological factors such as variable ctDNA shedding, low tumor burden, or disease distribution, as well as differences in analytical sensitivity between the two assays. Conversely, the high NPA (90.9%) indicates a high level of agreement for *KRAS* wild-type results. Importantly, the PABAK estimate (0.667) was similar to the observed Cohen’s kappa, suggesting that adjustment for prevalence and bias did not substantially alter the estimate of agreement. Nevertheless, these findings should be interpreted cautiously given the limited sample size. The two plasma-positive/tissue-negative cases may have several possible explanations, including spatial tumor heterogeneity not captured by single-lesion tissue sampling, subclonal alterations present in other tumor sites, or a hematopoietic origin of the circulating variant. In particular, clonal hematopoiesis of indeterminate potential (CHIP) represents an important potential confounder in plasma-based molecular analyses. Because matched white blood cell sequencing was not performed in the present study, a hematopoietic origin of these plasma-only *KRAS* alterations cannot be excluded. Therefore, these cases should not be considered definitive false-positive results, and their interpretation requires caution. Prospective studies incorporating matched tumor, plasma, and leukocyte sequencing will be important to establish the biological origin of plasma *KRAS* alterations.

The agreement in mutation subtype among paired positive cases was high, although not complete. A potential source of discordance between tissue and plasma mutational status lies in the inherent technical differences between the testing platforms. While NGS allows high-throughput identification and quantification of VAF in tissue, the Biocartis Idylla™ ctDNA system relies on automated real-time PCR. Although Idylla™ offers a rapid turnaround time and high analytical sensitivity, low-level circulating tumor DNA fragments below the analytical detection threshold may yield false-negative plasma results. In clinical practice, low ctDNA shedding (potentially associated with low tumor burden, specific metastatic sites, or slower-growing lesions) may further contribute to discordance. Future studies incorporating quantitative ultra-deep NGS or digital droplet PCR (ddPCR) could better characterize low-VAF alterations in plasma and help distinguish biological non-shedding from limitations related to assay sensitivity.

The strong quantitative correlation between tissue VAF and plasma molecular levels further supports an association between tissue mutational burden and circulating KRAS levels. However, this relationship should not be interpreted as evidence that plasma ctDNA quantitatively reproduces tissue tumor burden in all patients, given the biological and anatomical factors affecting ctDNA release. Rather, the observed correlation suggests that, when detectable, plasma *KRAS* levels may provide complementary quantitative information on the underlying tumor molecular profile.

While high concordance was observed between tissue and plasma *KRAS* status, the identification of plasma-positive/tissue-negative alterations provides an important illustration of the biological complexity of liquid biopsy. Such discrepancies may reflect spatial tumor heterogeneity or subclonal alterations that are not represented in the sampled tissue lesion. Alternatively, as noted above, a hematopoietic origin related to clonal hematopoiesis cannot be excluded in the absence of matched leukocyte sequencing. Therefore, plasma-only *KRAS* alterations should not be interpreted unequivocally as evidence of tumor evolution or emerging resistance. In the present study, the data support the potential of liquid biopsy to provide complementary molecular information beyond that obtained from a single tissue specimen but do not establish its ability to identify tumor evolution in real time.

In addition, longitudinal monitoring revealed dynamic changes in plasma *KRAS* status in selected patients. In some cases, changes in *KRAS*-mutated ctDNA occurred before clinically documented disease progression, whereas decreases in plasma *KRAS* signal were observed in patients subsequently achieving clinical benefit. These observations are consistent with the hypothesis that longitudinal ctDNA monitoring may capture molecular changes occurring during treatment. However, the longitudinal analysis included only nine patients, no prospectively defined criteria for molecular response or progression were applied, and treatment decisions were not systematically based on ctDNA findings. Therefore, these observations should be regarded as exploratory and hypothesis-generating rather than as evidence of clinical utility or treatment guidance.

The observed concordance between tissue and plasma *KRAS* status is consistent with previous reports in advanced lung cancer, where agreement may be influenced by biological and technical factors. In our cohort, the slightly lower detection rate of *KRAS* mutations in plasma compared with tissue (30.0% vs. 38.89%) likely reflects variability in ctDNA shedding, tumor burden, disease biology, and assay sensitivity. Although most patients presented with multiple metastatic sites, ctDNA detectability remained heterogeneous, suggesting that factors beyond the extent of disease may influence the release and persistence of tumor-derived DNA in the circulation.

Along with the concordance in mutation detection, the high agreement in mutation subtype among paired positive cases is relevant to the analytical characterization of plasma *KRAS* alterations. All detected alterations involved codon 12, with G12C being the predominant variant in both tissue and plasma. These findings support the analytical feasibility of plasma-based *KRAS* assessment when ctDNA is detectable, although the limited number of positive paired cases prevents broader conclusions regarding the performance of individual mutation subtypes.

No significant associations were observed between *KRAS* mutational status or expression levels and clinicopathological variables. While some non-significant trends emerged, these findings likely reflect both the limited sample size and the intrinsic heterogeneity of *KRAS*-mutant lung cancer.

A relevant finding of this study was the association between plasma *KRAS* positivity and survival. Patients with detectable *KRAS* mutations in plasma exhibited shorter OS and PFS, suggesting that plasma *KRAS* positivity may identify a subgroup of patients with less favorable clinical outcomes. However, given the limited cohort size and the small number of events, these findings should be considered exploratory. In particular, the multivariable Cox models were underpowered to establish an independent prognostic effect of plasma *KRAS* status, and the non-significant adjusted associations should not be interpreted as evidence of independent prognostic value. Larger prospective cohorts are required to determine whether plasma *KRAS* status provides prognostic information beyond established clinical variables.

From a clinical perspective, liquid biopsy represents a potentially valuable complementary approach to tissue-based molecular profiling. Tissue analysis remains essential for molecular characterization but is limited by procedural constraints, sampling of a single tumor site, and the inability to repeatedly characterize tumor molecular changes over time. In contrast, ctDNA analysis enables minimally invasive serial sampling and may provide complementary information on molecular changes during treatment. However, the present study does not demonstrate that ctDNA monitoring improves clinical decision-making or should replace tissue-based assessment.

Beyond mutation-based analysis, emerging approaches in fragmentomics may provide additional biological information from circulating cell-free DNA. Mechanistic fragmentomics seeks to link cfDNA fragment characteristics to the biological processes underlying DNA generation, release, fragmentation, and clearance. Apoptosis, necrosis, active cellular secretion, nuclease activity, and tissue-specific differences in chromatin organization may influence cfDNA abundance and fragment characteristics, potentially providing information on the cellular and tissue sources contributing to the circulating DNA pool [36,37,38]. This framework may be particularly relevant to liquid biopsy studies in which the biological origin of circulating variants is uncertain. Fragmentomic features, potentially integrated with genomic and epigenomic information, may provide complementary evidence for distinguishing tumor-derived cfDNA from hematopoietic cfDNA and could therefore help address confounding factors such as clonal hematopoiesis [36,38]. Although fragmentomic features were not investigated in the present study, integration of mutation-based ctDNA analysis with mechanistic fragmentomics represents a promising direction for future studies aimed at improving the biological specificity and clinical interpretation of liquid biopsy [38,39].

This pilot study has several limitations. The small sample size and single-center design limit statistical power and generalizability. The inclusion of heterogeneous histologies and treatment regimens, together with the limited number of patients undergoing longitudinal monitoring, may have influenced the observed results. In addition, differences in analytical sensitivity between tissue and plasma assays and the lack of systematic integration of tumor burden metrics may have affected concordance and interpretation of ctDNA dynamics. The absence of matched WBC sequencing also prevents definitive assessment of the potential contribution of clonal hematopoiesis to plasma-only *KRAS* alterations. Furthermore, the longitudinal analysis was exploratory, with no prospectively defined criteria for molecular progression or response and no systematic use of ctDNA results to guide treatment decisions. Finally, the limited number of survival events restricts the reliability of multivariable prognostic modelling. These limitations should be considered when interpreting the observed associations and reinforce the need for validation in larger prospective cohorts.

## 5. Conclusions

Our pilot study demonstrates that *KRAS* mutations detected in ctDNA are associated with less favorable survival outcomes in metastatic lung cancer and that plasma-based KRAS assessment shows substantial observed agreement with tissue genotyping. Longitudinal changes in plasma *KRAS* were observed in selected patients and, in some cases, preceded clinically documented changes in disease status. However, these findings are exploratory and do not establish ctDNA as an independent prognostic biomarker, a validated marker of real-time tumor evolution, or a tool for treatment guidance.

Despite the limited cohort size, the findings support further investigation of plasma-based *KRAS* assessment as a complementary approach to tissue molecular profiling. Future prospective studies incorporating larger cohorts, standardized longitudinal sampling, matched leukocyte sequencing, and integration of genomic and mechanistic fragmentomic features will be important to establish the biological specificity, prognostic value, and potential clinical utility of ctDNA-based monitoring in metastatic lung cancer.

## Figures and Tables

**Table 1:** Demographic and clinical features of the patients enrolled in the study (*n* = 40). Data are presented as number of patients and percentage. For metastatic sites at enrolment, patients with a single metastatic site are reported according to the specific anatomical location, whereas patients presenting with two or more metastatic sites are included in the “Multiple” category. Categories are mutually exclusive.

Feature		Number of Patients (%)
Gender	Male	25 (62.50)
	Female	15 (37.50)
Histology	Adenocarcinoma	24 (60.00)
	Squamous carcinoma	8 (20.00)
	Other	8 (20.00)
Number of metastatic sites at enrolment	1	4 (10.00)
	2	16 (40.00)
	3	13 (32.50)
	4	7 (17.50)
Metastatic sites at enrolment	Lymph nodes	2 (5.00)
	Bone	3 (7.50)
	Liver	1 (2.50)
	Lung	1 (2.50)
	Brain	1 (2.50)
	Pleura	1 (2.50)
	Adrenal gland	1 (2.50)
	Multiple	30 (75.00)
Surgery for primary tumor	No	32 (80.00)
	Yes	6 (15.00)
	Undefined	2 (5.00)

**Table 2:** Contingency table of the concordance analysis between *KRAS* mutational status in tissue and plasma. (A) Data are presented as absolute patient counts. PPA (Positive Percent Agreement) = 71.4% (95% CI: 45.4–88.3%); NPA (Negative Percent Agreement) = 90.9% (95% CI: 72.2–97.5%); OPA (Overall Percent Agreement) = 83.3% (95% CI: 68.1–92.1%). Inter-assay concordance: Cohen’s *κ* = 0.640 (95% CI: 0.377–0.903). (B) PPA, NPA, PPV, NPV, and OPA confidence intervals were computed using the Wilson score method. Cohen’s kappa standard error was calculated using Fleiss’ formula. PABAK (prevalence- and bias-adjusted kappa) was additionally calculated to account for the potential influence of the marginal distribution of positive and negative results. Spearman’s rank correlation coefficient ρ was derived from 29 patients with available quantitative molecular data.

(A) Matched 2 × 2 Contingency Matrix (*n* = 36)			
	**FFPE Tissue Mutated (+)**	**FFPE Tissue Wild-Type (−)**	Total
Plasma ctDNA Mutated (+)	10	2	12
Plasma ctDNA Wild-Type (−)	4	20	24
Total	14	22	36
(B) Concordance and Performance Metrics			
**Metric/Analysis**	**Estimate**	**95% Confidence Interval**	***p* Value/Clinical Interpretation**
Positive Percent Agreement (PPA)	71.4%	45.4–88.3%	Agreement for *KRAS* mutated cases
Negative Percent Agreement (NPA)	90.9%	72.2–97.5%	Agreement for *KRAS* wild-type cases
Positive Predictive Value (PPV)	83.3%	55.2–95.3%	Proportion of plasma- and tissue-positive cases
Negative Predictive Value (NPV)	83.3%	64.1–93.3%	Proportion of plasma- and tissue-negative cases
Overall Percent Agreement (OPA)	83.3%	68.1–92.1%	Overall observed agreement
Cohen’s Kappa (κ)	0.640	0.377–0.903	Substantial agreement
**PABAK**	**0.667**	—	Prevalence- and bias-adjusted agreement
**McNemar’s Exact Test**	—	—	*p* = 0.6875 (No statistically significant marginal asymmetry detected)
**Tissue VAF vs. Plasma Correlation (** n=29 **)**	ρ = 0.7757	0.5335–0.8824	*p* < 0.0001 (Strong positive correlation)

**Table 3:** Longitudinal plasma *KRAS* mutational status, ΔCq values, treatment, clinical response, and clinical course in mLC patients undergoing serial monitoring. Mt: mutated; Wt: wild type; END: end of the study; PR: partial response; SD: stable disease; PD: progressive disease. ΔCq values are reported when available. Longitudinal molecular changes are presented descriptively and do not represent prospectively defined criteria of molecular response or progression. Clinical course and treatment changes are reported descriptively. Treatment decisions were based on standard clinical and/or radiological assessment and were not prospectively guided by ctDNA findings.

	Baseline	4 Weeks	8 Weeks	12 Weeks	24 Weeks	Therapy	Best Response	Clinical Course and Treatment Changes
**CaPoS001**	Mt (G12V, ΔCq = 7.11)	M (G12V, ΔCq = 11.72)	**END**			Nivolumab + Ipilimumab + Carboplatin + Pemetrexed	PD	Early death during treatment
**CaPoS002**	Wt	Wt	Wt	**END**		Cemiplimab	PD	Clinical progression; treatment switched to second-line therapy
**CaPoS003**	Wt	Wt	Mt (G12D, ΔCq = 6.67)	Mt (G12D, ΔCq = 8.44)	Mt (G12D, ΔCq = 6.27)	Nivolumab + Ipilimumab + Carboplatin + Pemetrexed	PD	Clinical progression; treatment switched to second-line therapy
**CaPoS004**	Mt (G12C, ΔCq = 6.01)		Mt (G12C, ΔCq = 3.50; G12R, ΔCq = 14.81)	**END**		Carboplatin + Pemetrexed + Pembrolizumab followed by Pembrolizumab + Pemetrexed	PD	Clinical progression; treatment switched to second-line therapy
**CaPoS005**	Wt	Mt (G12R, ΔCq = 10.7)	Wt	Wt	Wt	Nivolumab + Ipilimumab + Carboplatin + Pemetrexed	PD	Mixed radiological response; subsequent treatment switched to second-line therapy based on clinical/radiological assessment
**CaPoS007**	Wt	Wt	Mt (G12V, ΔCq = 15.91)		**END**	Nivolumab + Ipilimumab + Carboplatin + Paclitaxel	PD	Clinical progression; treatment switched to second-line therapy
**CaPoS009**	Mt (G12S, ΔCq = 6.37)	Wt	Mt (G12S, ΔCq = 10.06)	Wt		Carboplatin + Pemetrexed + Pembrolizumab	PR	Mixed radiological response; subsequent treatment switched to second-line therapy based on clinical/radiological assessment
**CaPoS012**	Wt	Wt	Wt			Nivolumab + Ipilimumab + Carboplatin + Gemcitabine	PR	Mixed radiological response; subsequent treatment switched to second-line therapy based on clinical/radiological assessment
**CaPoS015**	Mt (G12C, ΔCq = 7.8)	Mt (G12C, ΔCq = 8.67)	Mt (G12C, ΔCq = 8.29)	Mt (G12C, ΔCq = 7.14)	END	Nivolumab + Ipilimumab + Carboplatin + Pemetrexed	SD	Clinical progression; treatment switched to second-line therapy

**Table 4:** Univariate and Multivariate Cox Proportional Hazards Analysis for OS and PFS.

Clinical Variable	Progression-Free Survival (PFS)		Overall Survival (OS)	
	Adjusted HR (95% CI)	*p* Value	Adjusted HR (95% CI)	*p* Value
**ctDNA KRAS status** (Mutated vs. WT)	1.902 (0.741–4.731)	0.176	1.770 (0.528–5.677)	0.346
**Age** (continuous, per year)	0.994 (0.941–1.051)	0.829	1.041 (0.961–1.147)	0.376
**Sex** (Male vs. Female)	1.233 (0.515–2.992)	0.636	3.892 (0.998–25.69)	0.101

## Data Availability

The datasets generated and analyzed during the present study are available from the corresponding author upon reasonable request.

## Supplementary Materials

The following supporting information can be downloaded at: https://www.mdpi.com/article/10.3390/cancers18183009/s1, Figure S1. Representative amplification curves obtained with Biocartis Idylla. (A) G12V mutated sample. (B) Sample bearing a G12S mutation. Figure S2. Survival analysis. Kaplan Meier plots for TTP. Black curve: wild type *KRAS* patients; Red curve: mutated *KRAS* patients. Table S1. Schedules of therapy for the patients enrolled in the study.
