# Supplementary material for: Dynamic Plasma KRAS ctDNA Profiling Identifies Treatment Response and Nascent Resistance in Metastatic Non-Small-Cell Lung Cancer: A Pilot Study
Source: Cancers (Basel). 2026 Sep 16;18(18):3009. doi: 10.3390/cancers18183009 (PMC13604566; doi:10.3390/cancers18183009)
Supplement: Supplementary file 1 [file cancers-18-03009-s001.zip › cancers-4540927-supplementary.pdf]

SUPPLEMNTARY INFORMATION

Dynamic Plasma KRAS ctDNA Profiling Identifies Treatment Response and Nascent Resistance in Metastatic Non-Small-Cell Lung Cancer: A Pilot Study

Valentina Buzzi [1,2], Greta Berti [3,4], Michele Santillo [1,2], Arianna Belli [5], Elisabetta Rosi [5], Giulia Favarato [6], Federico Lavorini [1,5], Sara Tomassetti [1,6,†], Annarosa Arcangeli [1], Mauro Iannopollo [3,4] and Elena Lastraioli [1,*]

[1] Department of Experimental and Clinical Medicine, University of Florence, 50121 Florence, Italy; v.buzzi@student.unisi.it (V.B.); michele.santillo@unifi.it (M.S.); federico.lavorini@unifi.it (F.L.); sara.tomassetti@auslromagna.it (S.T.); annarosa.arcangeli@unifi.it (A.A.)

[2] Department of Medical Biotechnologies, University of Siena, 53100 Siena, Italy

[3] Medical Oncology, S. Jacopo Hospital, 51100 Pistoia, Italy; greta.berti@uslcentro.toscana.it (G.B.)

[4] Medical Oncology, S.S. Cosma e Damiano Hospital, 51017 Pescia, Italy

[5] Pulmonology and Intensive Care Unit, Careggi University Hospital, 50134 Florence, Italy; arianna.belli@unifi.it (A.B.); rosiel@aou-careggi.toscana.it (E.R.)

[6] Interventional Pulmonology Unit, Careggi University Hospital, 50134 Florence, Italy; giulia.favarato@unifi.it

*Correspondence: elena.lastraioli@unifi.it

†Current address: Department of Specialistic Medicine, Rimini and Riccione Hospitals, AUSL Della Romagna, 48121 Rimini, Italy.

Supplementary Figures

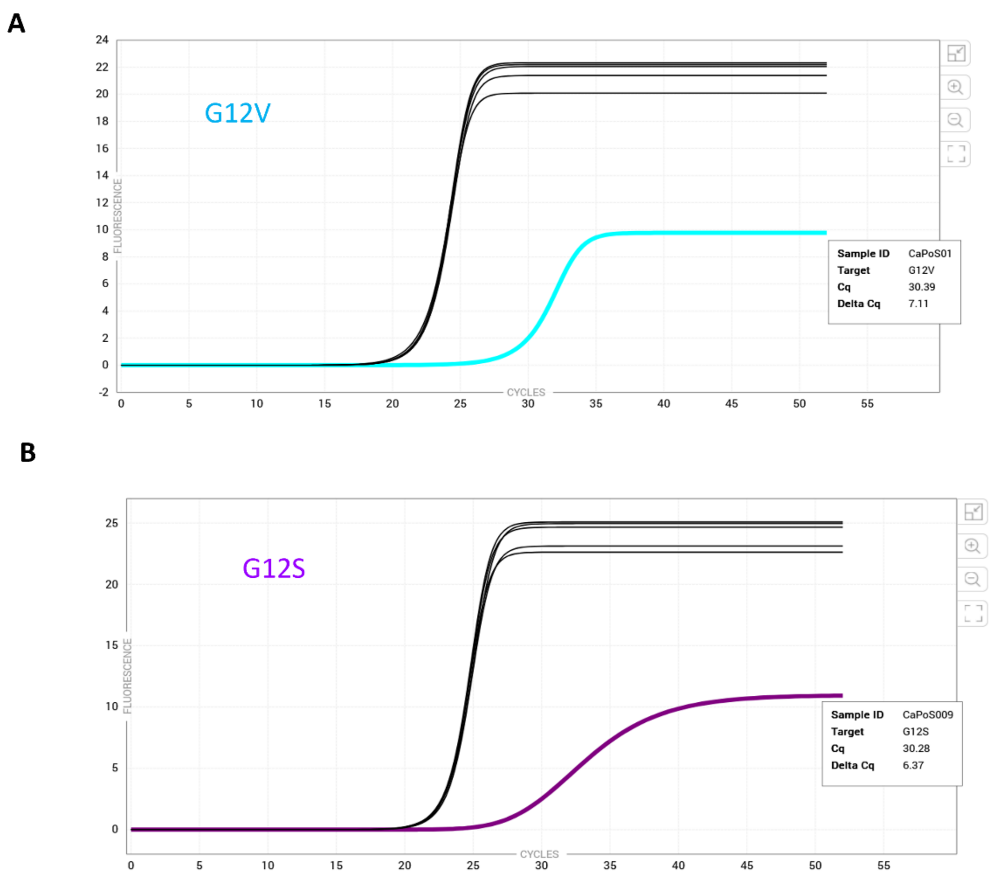

Figure S1. Representative amplification curves obtained with Biocartis Idylla. A) G12V mutated sample. B) Sample bearing a G12S mutation.

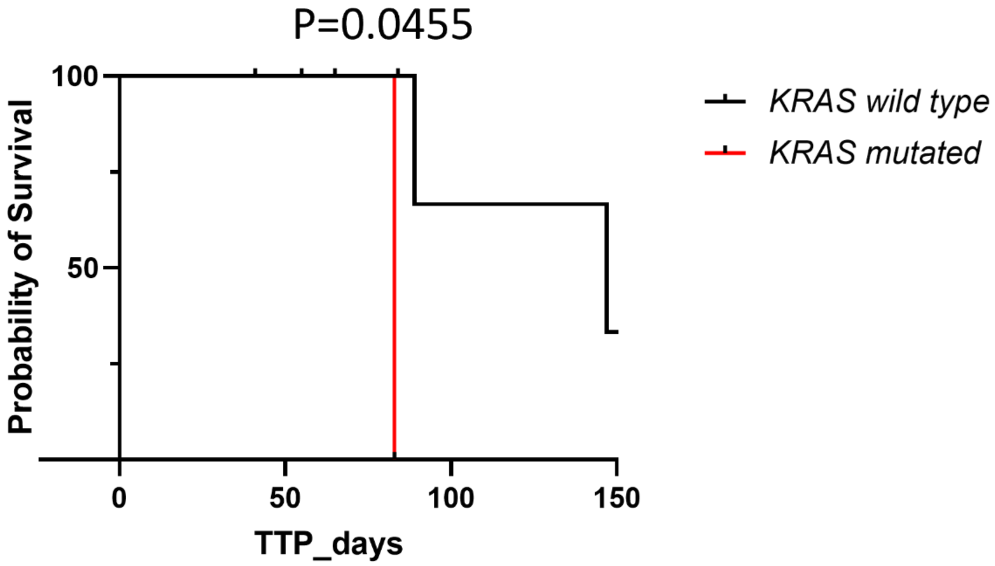

Figure S2. Survival analysis. Kaplan Meier plots for TTP. Black curve: wild type *KRAS* patients; Red curve: mutated *KRAS* patients.

Supplementary Table

Patient ID	Treatment regimen
CaPoS001	Carboplatin + Pemetrexed + Pembrolizumab + Adagrasib/Placebo
CaPoS002	Atezolizumab
CaPoS003	Gemcitabine
CaPoS004	Supportive care
CaPoS005	Carboplatin + Pemetrexed + Pembrolizumab
CaPoS006	Carboplatin + Pemetrexed
CaPoS007	Carboplatin + Pemetrexed + Nivolumab + Ipilimumab
CaPoS008	Carboplatin + Pemetrexed + Pembrolizumab
CaPoS009	Carboplatin + Etoposide + Atezolizumab
CaPoS010	Carboplatin + Pemetrexed + Amivantamab
CaPoS011	Carboplatin + Pemetrexed + Pembrolizumab for 4 cycles, followed by maintenance therapy with Pemetrexed + Pembrolizumab for up to 2 years
CaPoS012	Carboplatin + Paclitaxel + Rilvegostomig/Pembrolizumab
CaPoS013	Pembrolizumab + Pemetrexed + platinum-based chemotherapy
CaPoS014	Nivolumab + Ipilimumab + Carboplatin + Paclitaxel
CaPoS015	Carboplatin + Paclitaxel + Pembrolizumab
CaPoS016	Cemiplimab + Carboplatin + Paclitaxel
CaPoS017	Carboplatin + Pemetrexed + Nivolumab + Ipilimumab
CaPoS018	Managed/followed at another institution
CaPoS019	First-line chemo-immunotherapy with Carboplatin AUC 5 + Pemetrexed, followed by maintenance therapy with Pemetrexed + Pembrolizumab
CaPoS020	Gemcitabine
CaPoS021	Nivolumab + Ipilimumab + Carboplatin + Pemetrexed
CaPoS022	Cemiplimab
CaPoS AOUC 001	Nivolumab + Ipilimumab + Carboplatin + Gemcitabine
CaPoS AOUC 003	Atezolizumab
CaPoS AOUC 004	Carboplatin + Pemetrexed + Pembrolizumab
CaPoS AOUC 005	Carboplatin + Pemetrexed + Pembrolizumab for 4 cycles, followed by maintenance therapy with Pembrolizumab + Pemetrexed
CaPoS AOUC 006	Nivolumab + Ipilimumab + Carboplatin + Pemetrexed
CaPoS AOUC 007	Carboplatin + Pemetrexed + Pembrolizumab

CaPoS AOUC 008	Atezolizumab
CaPoS AOUC 010	Atezolizumab
CaPoS AOUC 011	Pemetrexed + Cemiplimab
CaPoS AOUC 012	Cemiplimab + Carboplatin + Gemcitabine
CaPoS AOUC 013	Nivolumab + Ipilimumab + Carboplatin + Pemetrexed
CaPoS AOUC 014	Carboplatin + Pemetrexed + Amivantamab (compassionate use)
CaPoS AOUC 015	Carboplatin + Pemetrexed + Amivantamab (compassionate use)
CaPoS AOUC 016	Nivolumab + Ipilimumab + Carboplatin + Oral Vinorelbine
CaPoS AOUC 017	Nivolumab + Ipilimumab + Carboplatin + Pemetrexed
CaPoS AOUC 019	Pembrolizumab + Carboplatin + Pemetrexed
CaPoS AOUC 020	Carboplatin AUC 5 + Pemetrexed + Pembrolizumab/Rilvegostomig
CaPoS AOUC 022	Carboplatin + Pemetrexed + Pembrolizumab

Table S1- Schedules of therapy for the patients enrolled in the study
